# Supplementary material for: Anti-Citrullinated Protein Antibody Reactivity towards Neutrophil-Derived Antigens: Clonal Diversity and Inter-Individual Variation
Source: Biomolecules. 2023 Mar 31;13(4):630. doi: 10.3390/biom13040630 (PMC10135477; doi:10.3390/biom13040630)
Supplement: Supplementary file 1 [file biomolecules-13-00630-s001.zip › biomolecules-2299173-supplementary.pdf]

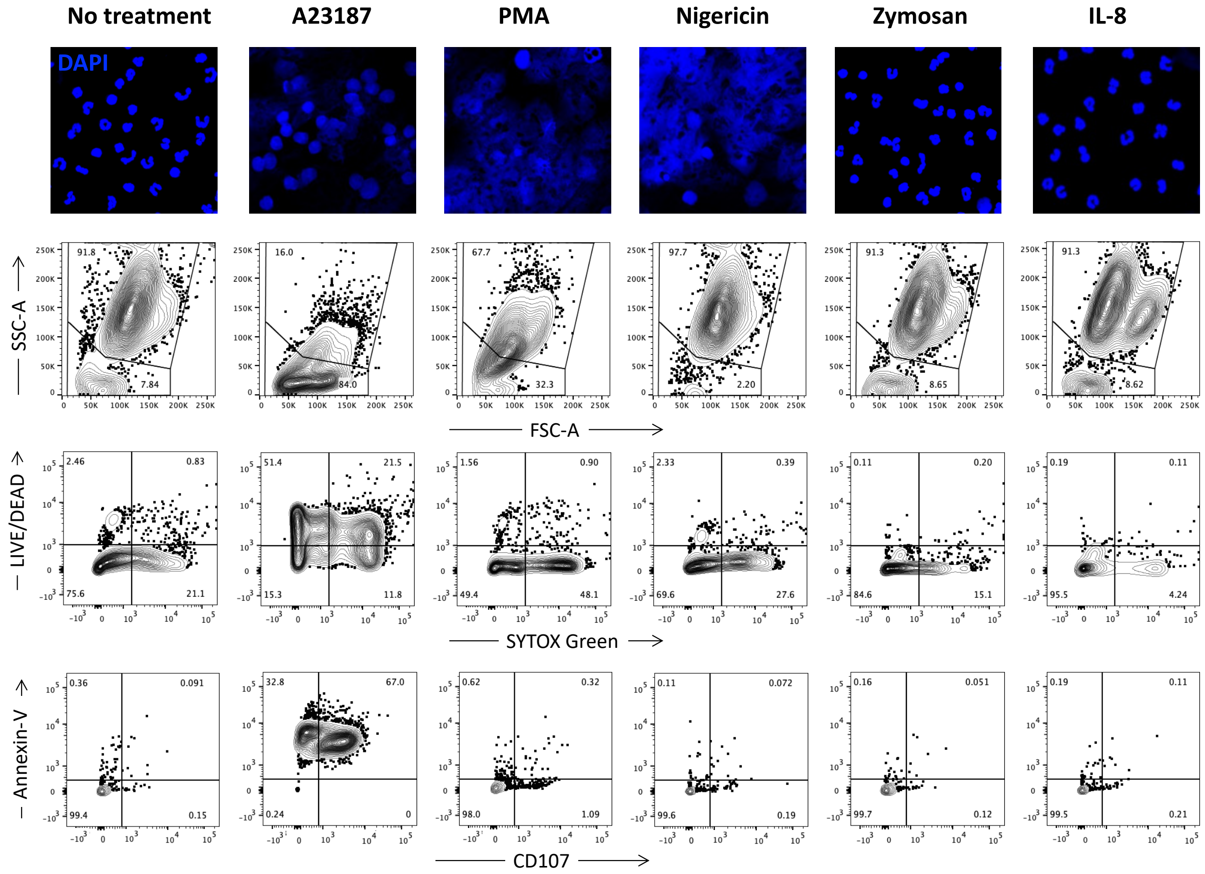

Supplementary figure 1. Detection of cell death and degranulation in neutrophils. Cells exposed to different stimuli for three hours were studied by confocal microscopy, using DAPI staining of the DNA, and flow cytometry where cell size and granularity were evaluated by measuring forward scatter (FSC) and side scatter (SSC), respectively. Cell death was analyzed by staining with Live/Dead, SYTOX green or Annexin-V dyes in non-fixed samples, degranulation was studied via CD107 staining. Representative results of three experiments are shown.

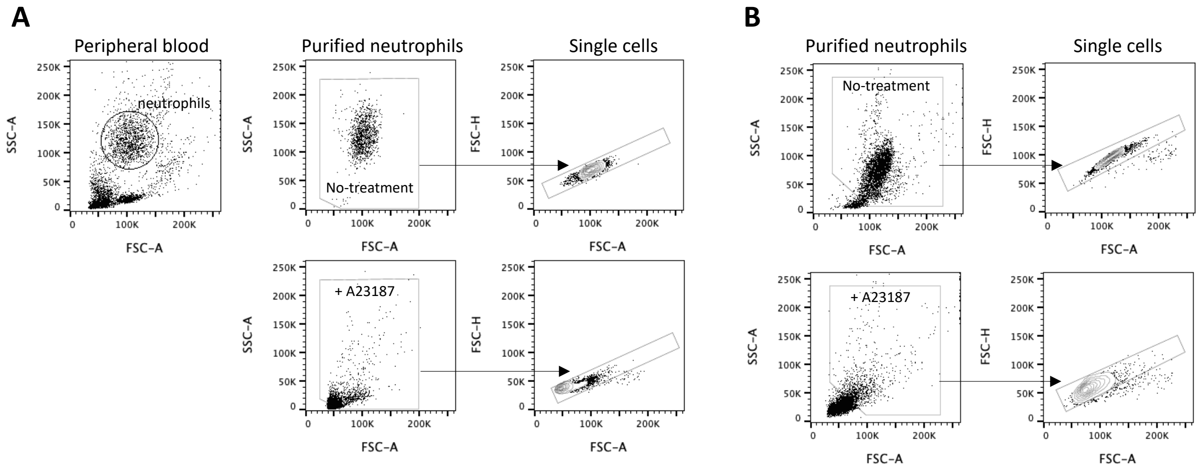

Supplementary figure 2. Neutrophil gating strategy for flow cytometry analyses. Relatively broad gates were used and only the smallest-size debris was excluded in the forward scatter area (FSC-A) – side scatter area (SSC-A) plots for both human (A) and murine (B) cell analyses, as the fixation-permeabilization protocol and the application of different activation stimuli had a major impact on cell morphology. Non-activated neutrophils could be distinguished due to their relatively high SSC-A levels as compared to other cell types.

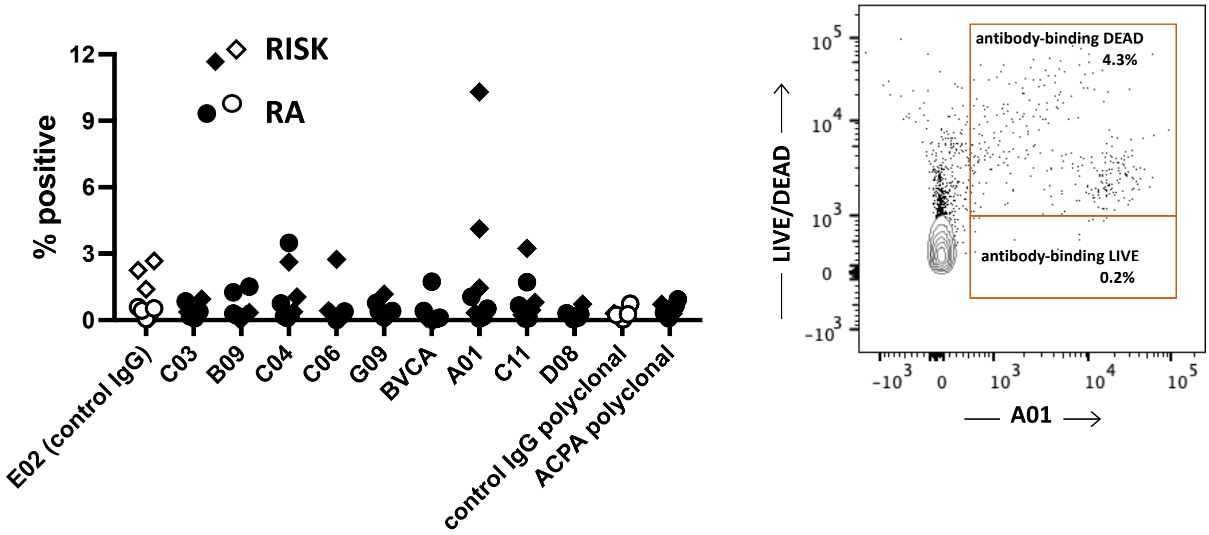

Supplementary figure 3. ACPA binding to neutrophils obtained from individuals at risk of RA or from patients with established RA

Neutrophils were isolated from peripheral blood samples and stained with different ACPA or control antibody preparations, symbols represent individual blood donors. A representative contour plot indicates that ACPA binding occurred to a small number of dead cells in the samples.

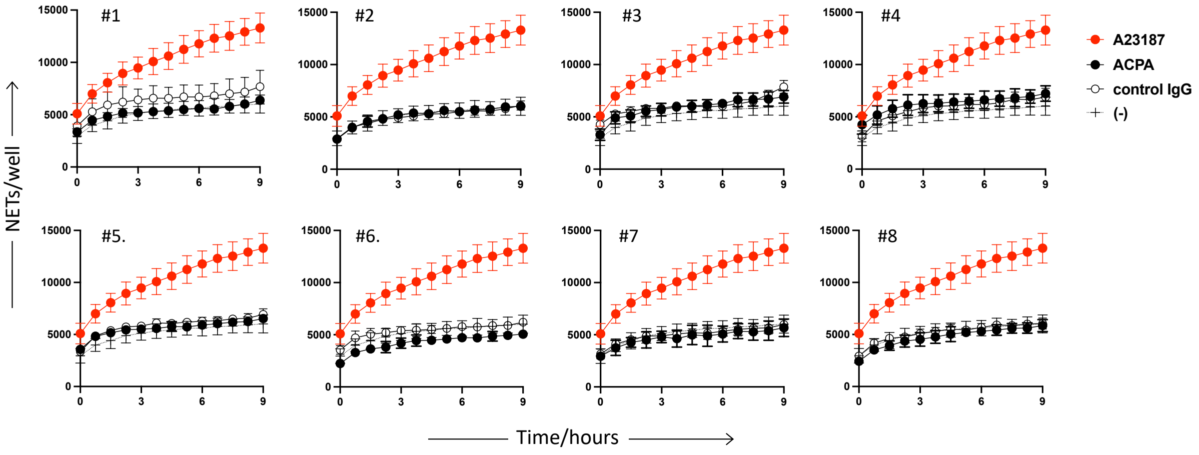

Supplementary figure 4. Analysis of NETosis in the presence of ACPA and control IgG preparations obtained from eight individual patients.

The antibodies were added to the cultures in 50μg/ml concentration, non-treated (-) and A23187-treated samples were used as negative and positive controls, respectively. NETosis was analyzed with IncuCyte Zoom, representative results of two independent experiments are shown.

Supplementary Table 1. Characteristics of the RA patients involved in the study

Individual ACPA preparations								
Sample	Age (years)	Sex	Time from diagnosis (years)	RF	ACPA	CRP (above cutoff)	Smoking	Treatment (at time of samples)
1	65	f	6	pos	pos	no	ever	MTX, ADA
2	60	f	44	pos	pos	yes	unknown	CS
3	84	m	4	neg	pos	yes	ever	MTX
4	72	f	<1	pos	pos	yes	never	MTX, CS
5	82	f	12	pos	pos	yes	never	Etanercept, CS
6	81	f	9	pos	pos	yes	never	SSZ, ADA, CS
7	68	f	9	pos	pos	no	ever	MTX
8	30	f	<1	pos	pos	no	current	SSZ, CS
FACS analyses								
9	49	f	1	pos	pos	no	ever	MTX, ADA
10	70	m	8	pos	pos	yes	ever	MTX, ADA, CS
11	77	f	1	pos	pos	no	never	MTX, RTX
12	76	f	3	pos	pos	yes	Ever	MTX

MTX - Methotrexate, ADA – Adalimumab, SSZ - Sulfasalazine, CS – corticosteroid (Prednisolon, ≤7.5mg daily)RTX - Rituximab

Supplementary Table 2. Modified peptides used for characterizing fine-specificities in monoclonal and polyclonal ACPA

Peptide	Protein chain, residues	Sequence*	Assay	Reference
Cit Fibα_{36-50}	Fibrinogen α-chain, 36-50	GP(**cit**)VVF(**cit**)HQSACKDSK	Array	[2, 3]
Cit Fib$\alpha_{36-50-Cit1}$	Fibrinogen α-chain, 36-50	GP(**cit**)VVERHQSACKNSL	Array	
Cit Fib$\alpha_{36-50-Cit2}$	Fibrinogen α-chain, 36-50	GPRVVE(**cit**)HQSACKNSL	Array	
Cit Fibβ_{36-52}	Fibrinogen β-chain, 36-52	NEEGFFSA(**cit**)GHRPLDKK	Array	[4]
Cit Fibβ_{60-74}	Fibrinogen β-chain, 60-74	(**cit**)PAPPPISGGGY(**cit**)A(**cit**)	Array	[2, 3]
Cit Fib$\beta_{60-74-Cit1}$	Fibrinogen β-chain, 60-74	(**cit**)PAPPPISGGGYRAR	Array	
Cit Fib$\beta_{60-74-Cit2}$	Fibrinogen β-chain, 60-74	RPAPPPISGGGY(**cit**)AR	Array	
Cit Fib$\beta_{60-74-Cit3}$	Fibrinogen β-chain, 60-74	RPAPPPISGGGYRA(**cit**)	Array	
Cit Fil$_{307-324}$	Filaggrin, 307-324	HQCHQEST(**cit**)GRSRGRCGRSGS[cyclic]	Array	[5]
Carb Fil$_{307-324}$	Filaggrin, 307-324	HQCHQEST(**carb**)GRSRGRCGRSGS[cyclic]	Array	[5]
Acet Fil$_{307-324}$	Filaggrin, 307-324	HQCHQEST(**acet**)GRSRGRCGRSGS[cyclic]	Array	[5]
Cit H3$_{1-30}$	Histone 3, 1-30	A(**cit**)TKQTA(**cit**)KSTGGKAP(**cit**)KQLATKAA(**cit**)KSAP	Array	[6, 7]
Cit H3$_{21-44}$	Histone 3, 21-44	ATKAA(**cit**)KSAPATGGVKKPH(**cit**)Y(**cit**)PGGGK	Array	[7]
Cit TNC1	Tenascin, 2026-2040	VFLRRKNG(**cit**)ENFYQNW	Array	[8]
Cit TNC5	Tenascin, 2176-2200	EHSSIQFAEMKL(**cit**)PSNF(**cit**)NLEG(**cit**)(**cit**)K(**cit**)	Array	[8]
Cit TNC5$_{Cit1}$	Tenascin, 2176-2200	EHSIQFAEMKL(**cit**)PSNFRNLEGRRKR	Array	
Cit TNC5$_{Cit2}$	Tenascin, 2176-2200	EHSIQFAEMKLRPSNF(**cit**)NLEGRRKR	Array	
Cit TNC5$_{Cit3}$	Tenascin, 2176-2200	EHSIQFAEMKLRPSNFRNLEG(**cit**)(**cit**)KR	Array	
Cit TNC5$_{Cit4}$	Tenascin, 2176-2200	EHSIQFAEMKLRPSNFRNLEG(**cit**)RKR	Array	
Cit TNC5$_{Cit5}$	Tenascin, 2176-2200	EHSIQFAEMKLRPSNFRNLEGR(**cit**)KR	Array	
Cit Vim$_{60-75}$	Vimentin, 60-75	VYAT(**cit**)SSAV(**cit**)L(**cit**)SSVP	Array	[4]
Cit Vim$_{60-75-Cit1}$	Vimentin, 60-75	VYAT(**cit**)SSAVRLRSSVP	Array	
Cit Vim$_{60-75-Cit2}$	Vimentin, 60-75	VYATRSSAV(**cit**)LRSSVP	Array	
Cit Vim$_{60-75-Cit3}$	Vimentin, 60-75	VYATRSSAVRL(**cit**)SSVP	Array	
Acet His4$_{1-18\ K5}$	Histone 4, 1-18	SGRG(**acet**)GGKGLGKGGAKRH	Array	[9]
Acet His4$_{1-18\ K16}$	Histone 4, 1-18	SGRGKGGKGLGKGGA(**acet**)RH	Array	[9]
Cit-His4$_{1-18\ R3}$	Histone 4, 1-18	SG(**cit**)GKGGKGLGKGGAKRH	ELISA	[10]
Acet His2B$_{6-22}$	Histone 2B, 6-22	HQCSAPAPK(**acet**)GSKKAVTKAQC[cyclic]	ELISA	[9]
Cit-His4$_{14-34}$	Histone 4, 14-34	GAK(**cit**)H(**cit**)KVL(**cit**)DNIQGITKPAI	ELISA	[11]
Cit hnRNPA1$_{199-212}$	hnRNP A1, 199-212	HQCGNFGGG(**cit**)GGGFGGNC[cyclic]	ELISA	
Cit hnRNPA1$_{211-224}$	hnRNP A1, 211-224	HQCGNDNFG(**cit**)GGNFSGRC[cyclic]	ELISA	
Cit Mod-Vim$_{58-69}$	Vimentin, 58-69	GRVYAT(**cit**)SSAVR	ELISA	[12]
Carb Mod-Vim$_{58-69}$	Vimentin, 58-69	GRVYAT(**carb**)SSAVR	ELISA	[12]
Acet Mod-Vim$_{58-69}$	Vimentin, 58-69	GRVYAT(**acet**)SSAVR	ELISA	[12]

*The peptide amino acid sequence displayed where arginine (R) is substituted with citrulline in (cit). Both citrulline containing peptides and native arginine containing peptides were also analysed on the array.

In Fil$_{307-324}$ and Mod-Vim$_{58-69}$ the original arginine (R) was also substituted for carbamyl-lysine/homocitrulline (Carb) or acetyl-lysine (Acet). In the Acet-His2B/4 peptides the original lysine (K) residues were replaced with acetyl-lysine as indicated. All peptides were biotinylated when used in assays.

Besides native arginine peptides the following control autoantigens were included on the array: CENP B, collagen II, fibrillarin, Jo-1, Mi-2, PCNA, PM-Scl 100, Rip P0. Rip P1, Rip P2, RNA Pol III, RNP-70, RNP-A, RNP-C, Ro52, Ro60, Scl-70, SmBB, Sm, SSB/La

The ACPA fine-specificity array is further described in [12, 13].

References

[1] Sahlström P, Hansson M, Steen J, et al. Different Hierarchies of Anti–Modified Protein Autoantibody Reactivities in Rheumatoid Arthritis. Arthritis & Rheumatology 2020;72(10):1643-57.

[2] C. Iobagiu, A. Magyar, L. Nogueira, M. Cornillet, M. Sebbag, J. Arnaud *et al.* The antigen specificity of the rheumatoid arthritis-associated ACPA directed to citrullinated fibrin is very closely restricted. J Autoimmun, 2011;37:263-72.

[3] M. Sebbag, N. Moinard, I. Auger, C. Clavel, J. Arnaud, L. Nogueira *et al.* Epitopes of human fibrin recognized by the rheumatoid arthritis-specific autoantibodies to citrullinated proteins. Eur J Immunol, 2006;36:2250-63.

[4] K. N. Verpoort, K. Cheung, A. Ioan-Facsinay, A. H. van der Helm-van Mil, J. K. de Vries-Bouwstra, C. F. Allaart *et al.* Fine specificity of the anti-citrullinated protein antibody response is influenced by the shared epitope alleles. Arthritis Rheum, 2007;56:3949-52.

[5] G. A. Schellekens, H. Visser, B. A. de Jong, F. H. van den Hoogen, J. M. Hazes, F. C. Breedveld *et al.* The diagnostic properties of rheumatoid arthritis antibodies recognizing a cyclic citrullinated peptide. Arthritis Rheum, 2000;43:155-63.

[6] K. M. J. Janssen, M. J. de Smit, C. Withaar, E. Brouwer, A. J. van Winkelhoff, A. Vissink *et al.* Autoantibodies against citrullinated histone H3 in rheumatoid arthritis and periodontitis patients. J Clin Periodontol, 2017;44:577-84.

[7] J. Steen, B. Forsstrom, P. Sahlstrom, V. Odowd, L. Israelsson, A. Krishnamurthy *et al.* Recognition of Amino Acid Motifs, Rather Than Specific Proteins, by Human Plasma Cell-Derived Monoclonal Antibodies to Posttranslationally Modified Proteins in Rheumatoid Arthritis. Arthritis Rheumatol, 2019;71:196-209.

[8] A. Schwenzer, X. Jiang, T. R. Mikuls, J. B. Payne, H. R. Sayles, A.-M. Quirke *et al.* Identification of an immunodominant peptide from citrullinated tenascin-C as a major target for autoantibodies in rheumatoid arthritis. Annals of the rheumatic diseases, 2016;75:1876-83.

[9] K. A. Lloyd, G. Wigerblad, P. Sahlström, M. G. Garimella, K. Chemin, J. Steen *et al.* Differential ACPA Binding to Nuclear Antigens Reveals a PAD-Independent Pathway and a Distinct Subset of Acetylation Cross-Reactive Autoantibodies in Rheumatoid Arthritis. Front Immunol, 2019;9:3033.

[10] R. G. S. Chirivi, J. W. G. van Rosmalen, M. van der Linden, M. Euler, G. Schmets, G. Bogatkevich *et al.* Therapeutic ACPA inhibits NET formation: a potential therapy for neutrophil-mediated inflammatory diseases. Cellular & Molecular Immunology, 2021;18:1528-44.

[11] F. Pratesi, I. Dioni, C. Tommasi, M. C. Alcaro, I. Paolini, F. Barbetti *et al.* Antibodies from patients with rheumatoid arthritis target citrullinated histone 4 contained in neutrophils extracellular traps. Ann Rheum Dis, 2014;73:1414-22.

[12] C. P. Figueiredo, H. Bang, J. F. Cobra, M. Englbrecht, A. J. Hueber, J. Haschka *et al.* Antimodified protein antibody response pattern influences the risk for disease relapse in patients with rheumatoid arthritis tapering disease modifying antirheumatic drugs. Ann Rheum Dis, 2017;76:399-407.

[13] M. Hansson, L. Mathsson, T. Schlederer, L. Israelsson, P. Matsson, L. Nogueira *et al.* Validation of a multiplex chip-based assay for the detection of autoantibodies against citrullinated peptides. Arthritis Res Ther, 2012;14:R201.

[14] J. Ronnelid, M. Hansson, L. Mathsson-Alm, M. Cornillet, E. Reed, P. J. Jakobsson *et al.* Anticitrullinated protein/peptide antibody multiplexing defines an extended group of ACPA-positive rheumatoid arthritis patients with distinct genetic and environmental determinants. Ann Rheum Dis, 2018;77:203-11.
